# Supplementary material for: Social Processes of Young Adults’ Recovery and Identity Formation during Life-Disruptive Mental Distress—A Meta-Ethnography
Source: Int J Environ Res Public Health. 2023 Aug 25;20(17):6653. doi: 10.3390/ijerph20176653 (PMC10487737; doi:10.3390/ijerph20176653)
Supplement: Supplementary file 1 [file ijerph-20-06653-s001.zip › ijerph-2495399-supplementary.pdf]

The Social Process of Youth Recovery and Identity Formation during Early Psychosis – a Meta-ethnography

Supplementary Materials:

Table S1: Controlled headings and free-text search terms included in the building block strategy in Embase.

Youth	Recovery	Psychosis	Identity	Qualitative methodology
Adolescent/<br>Young Adult/<br>young.ti.<br>youth.ti.<br>"adolescen*".ti.<br>"emerging adult*".ti.<br>"early adult*".ti.<br>"teen*".ti.<br>juvenile.ti.	mental health recovery/ or psychiatric rehabilitation/ or rehabilitation, vocational/ or self care/ or self-management/<br>"Quality of Life"/<br>personal satisfaction/<br>"Value of Life"/<br>emotional adjustment/ or survivorship/ or posttraumatic growth, psychological/<br>Health Knowledge, Attitudes, Practice/<br>exp family/ or intergenerational relations/ or social skills/<br>peer influence/<br>exp Social Networking/<br>Resilience, Psychological/<br>empowerment/<br>self efficacy/<br>exp Adaptation, Psychological/<br>"recover*".ab,ti,tw.<br>(psychosocial adj3 (adapt* or adjust* or develop* or rehabilitation or remission)).ab,ti,tw.<br>(mental adj3 (adapt* or adjust* or develop* or rehabilitation or remission)).ab,ti,tw.<br>(quality adj2 life).ab,ti,tw.<br>(value adj2 life).ab,ti,tw.<br>wellbeing.ab,ti,tw.<br>"well being".ab,ti,tw.<br>"empower*".ab,ti,tw.<br>"resilien*".ab,ti,tw.<br>coping.ab,ti,tw.<br>"health belie*".ab,ti,tw.<br>meaningful.ab,ti,tw.<br>(emotional adj3 (adapt* or adjust* or develop*)).ab,ti,tw.<br>"post traumatic growth".ab,ti,tw.<br>"posttraumatic growth".ab,ti,tw.<br>"sense of coherence".ab,ti,tw.<br>"life satisfaction".ab,ti,tw.<br>(self adj3 (care or effic* or manage*)).ab,ti,tw.	"schizophrenia spectrum and other psychotic disorders"/ or exp psychotic disorders/ or exp schizophrenia/<br>psychosis.ab,ti,tw.<br>psychotic.ab,ti,tw.<br>(psycho* adj3 onset).ab,ti,tw.<br>"early psycho*".ab,ti,tw.<br>"first episode of psychosis".ab,ti,tw.<br>"first episode psychosis".ab,ti,tw.<br>FEP.ab,ti,tw.<br>"schizophrenia spectrum disorder*".ab,ti,tw.<br>"hallucina*".ab,ti,tw.<br>"delusion*".ab,ti,tw.<br>(hear* adj3 voice*).ab,ti,tw.<br>(risk adj5 psychosis).ab,ti,tw.	gender identity/<br>personality/ or self concept/ or body image/ or self-assessment/<br>social identification/ or social stigma/<br>personal narrative/<br>role/<br>mental competency/ or individuality/ or identification, psychological/ or identity crisis/ or individuation/<br>exp Personality/<br>social stigma/<br>exp Social Behavior/<br>"identit*".ab,ti,tw.<br>"social status".ab,ti,tw.<br>"personalit*".ab,ti,tw.<br>"stigma*".ab,ti,tw.<br>(self adj3 (worth or concept or esteem or image or percept* or evaluat* or regard or assess* or knowledge or present* or negotiate*)).ab,ti,tw.<br>agency.ab,ti,tw.<br>autonomy.ab,ti,tw.<br>introspection.ab,ti,tw.<br>"possible sel*".ab,ti,tw.<br>"future sel*".ab,ti,tw.<br>"body image*".ab,ti,tw.<br>"body awareness".ab,ti,tw.<br>"personal narrative*".ab,ti,tw.<br>"role*".ab,ti,tw.<br>"social identification".ab,ti,tw.<br>individuality.ab,ti,tw.<br>individuation.ab,ti,tw.<br>"life experience*".ab,ti,tw.	qualitative research/<br>Focus Groups/<br>Anthropology, Cultural/mt [Methods]<br>grounded theory/<br>biography/ or interview/<br>exp personal narrative/<br>qualitative.ab,ti,tw.<br>"grounded theory".ab,ti,tw.<br>"interview*".ab,ti,tw.<br>"focus group*".ab,ti,tw.<br>"content analys*".ab,ti,tw.<br>"ethnograph*".ab,ti,tw.<br>"biograph*".ab,ti,tw.<br>"subjective account*".ab,ti,tw.<br>(patient* adj3 (perspective* or perception* or experience*)).ab,ti,tw.<br>"narrative*".ab,ti,tw.

	family.ab,ti,tw.<br>families.ab,ti,tw.<br>"parent*".ab,ti,tw.<br>"extended famil*".ab,ti,tw.<br>"friend*".ab,ti,tw.<br>"peer*".ab,ti,tw.<br>(social adj3 (network* or interact*)).ab,ti,tw.<br>"interpersonal*".ab,ti,tw.			
